# Siponimod ameliorates experimental autoimmune neuritis

**DOI:** 10.1186/s12974-023-02706-z

**Published:** 2023-02-14

**Authors:** Takafumi Uchi, Shingo Konno, Hideo Kihara, Toshiki Fujioka

**Affiliations:** 1 Division of Neurology, Department of Internal Medicine, Toho University Graduate School of Medicine, Tokyo, Japan; 2 Department of Neurology, Toho University Ohashi Medical Center, 2-22-36 Ohashi, Meguro-Ku, Tokyo, 153-8515 Japan

**Keywords:** Guillain–Barré syndrome, Chronic inflammatory demyelinating polyneuropathy, Experimental autoimmune neuritis, Siponimod, S1PR1, S1PR5

## Abstract

**Background:**

Guillain–Barré syndrome (GBS) and chronic inflammatory demyelinating polyneuropathy (CIDP) are human autoimmune peripheral neuropathy. Besides humoral immunity, cellular immunity is also believed to contribute to these pathologies, especially CIDP. Sphingosine-1-phosphate receptor 1 (S1PR1) regulates the maturation, migration, and trafficking of lymphocytes. As of date, the therapeutic effect of sphingosine-1-phosphate receptor (S1PR) agonists on patients with GBS or CIDP remains unclear.

**Methods:**

To evaluate the effect of siponimod, an agonist of S1PR1 and S1PR5, on experimental autoimmune neuritis (EAN), an animal model of autoimmune peripheral neuropathy, was used. Lewis rats were immunized with 125 μg of synthetic peptide from bovine P2 protein. Rats in the siponimod group were orally administered 1.0 mg/kg siponimod and those in the EAN group were administrated the vehicle on days 5–27 post-immunization (p.i.) daily. The symptom severity was recorded daily. The changes in the expression of cytokines and transcription factors in the lymph nodes and cauda equina (CE) which correlate with the pathogenesis of EAN and recovery of injured nerve were measured using reverse transcription quantitative PCR. Histological study of CE was also performed.

**Results:**

Flaccid paralysis developed on day 11 p.i. in both groups. Siponimod relieved the symptom severity and decreased the expression of interferon-gamma and IL-10 mRNAs in lymph nodes and CE compared with that in the EAN group. The expression of Jun proto-oncogene (c*-Jun*) mRNA increased from the peak to the recovery phase and that of Sonic hedgehog signaling molecule (*Shh*) and Glial cell line-derived neurotrophic factor (*Gdnf*) increased prior to increase in c-Jun with no difference observed between the two groups. Histologically, siponimod also reduced demyelinating lesions and inflammatory cell invasion in CE.

**Conclusions:**

Siponimod has a potential to ameliorate EAN. Shh and Gdnf, as well as C-Jun played a significant role during the recovery of injured nerves.

**Supplementary Information:**

The online version contains supplementary material available at 10.1186/s12974-023-02706-z.

## Background

Guillain–Barré syndrome (GBS) and chronic inflammatory demyelinating polyneuropathy (CIDP) are autoimmune-mediated forms of peripheral neuritis. The main distinction between GBS and CIDP is that in GBS, symptoms tend to appear after a preceding infection and reach their peak within 4 weeks and rarely recur, whereas in CIDP, the course is chronic, lasting more than 2 months [1–3]. Recently, anti-ganglioside antibodies were detected in 50–60% of patients with GBS, indicating a crucial role of humoral immunity in GBS [1, 2]. The most common treatment for GBS is intravenous immunoglobulin therapy (IVIg) [4], but some cases have sequelae [5]. Although many aspects of antibody production, including antigen presentation, mechanisms of antibody production, and differences in antibody subclasses, remain unknown, clinical trials using complement C5 inhibitors, such as eculizumab, in combination with IVIg to control complement-mediated pathology are ongoing [6].

In CIDP, although specific pathogenic antibodies have not been identified, the pathogenesis is thought to involve cellular immunity, such as activated T lymphocytes and macrophages, besides the humoral immunity. Treatment for CIDP, such as with corticosteroids, plasmapheresis, and IVIg, is effective [3]. However, long-term administration of corticosteroids is inevitably associated with side effects such as infection, osteoporosis, and depression. The latter two treatments are only effective for a short-term duration and require repeated administration. The prognosis after 5 years of treatment for CIDP was reported to be only 26% of patients in complete remission, and 39% requiring continuous treatment [7]. Therefore, new treatment modalities are needed for CIDP as for GBS.

Experimental autoimmune neuritis (EAN) has been used as an animal model of human autoimmune-mediated peripheral neuritis in the development of therapies [8]. Interferon-gamma (IFN-γ)-producing autoreactive T lymphocytes induce EAN through the migration of macrophages from lymph nodes (LN) to immune-target organs, such as the peripheral nerves. There are some similarities in the pathological findings of peripheral nerves in CIDP and EAN, in addition to cellular immunity-dominant pathogenesis. The pathology of peripheral nerves in CIDP is characterized by a mild T-cell infiltration, with macrophages being the predominant cells invading the endoneurium. Macrophages are the final effector cells involved in the mechanism of demyelination in CIDP [9]. EAN also has multifocal and often perivascular mononuclear cell infiltration, which involves, lymphocytes and peripheral nerve demyelination by macrophages [8].

In cellular immunity, a sphingosine 1-phosphate (S1P) gradient is formed due to a lower concentration of S1P in the lymphoid tissue than that in the efferent lymph vessels. The egress of lymphocytes from LN is dependent on the S1P gradient. S1P receptor 1 (S1PR1) expressed on naïve T cells responds to this gradient. When naïve T cells are activated by antigen-expressing dendritic cells, the expression of S1PR1 is reduced. They differentiate into effector cells in the T cell zone, re-expressing S1PR1, and can exit the LN [10].

A non-selective S1PR (1–5) agonist, fingolimod, and a functional S1PR1 antagonist and S1PR5 agonist, siponimod, have been approved as treatments for multiple sclerosis (MS). MS is a neuroinflammatory disease in which leukocytes infiltrate the central nervous system (CNS), activating microglia and astrocytes and causing axonal loss [11–13]. Fingolimod and siponimod act on the S1PR1 present on the surface of autoreactive T cells. S1PR1 bound by these therapeutic agents undergoes sustained cellular uptake and subsequent degradation, unlike S1P-bound S1PR1. Thus, autoreactive T cells from patients taking these MS drugs cannot respond to S1P, inhibiting its entry into the CNS and suppressing MS. Fingolimod has also been reported to be effective in treating EAN via inhibition of lymphocytes or/and through the infiltration of monocytes into the peripheral nerves [14–16].

The effectiveness of siponimod for EAN remains unknown. Herein, we describe the results of our evaluation of the potential of siponimod for the treatment of EAN and discuss molecular effects via S1PR1 and S1PR5.

## Materials and methods

### Rats

Female Lewis rats aged 7 weeks were purchased from the Charles River Japan (Yokohama, Japan). They were kept in the Toho University Ohashi Experimental Animal Laboratory according to the regulation and guidelines of the Toho University's Animal Experiment Committee. Every effort was made to minimize the suffering of rats.

### Induction and evaluation of EAN

The rats were subcutaneously injected with 125 µg of a synthetic peptide corresponding to the amino acid residues 53–78 of bovine P2 protein (TESPFKNTEISFKLGQEFEETTADNR, Operon, Tokyo, Japan), emulsified in an equal volume of Complete Freund's adjuvant (Sigma–Aldrich, MO, USA) in the right footpad [17] under light anesthesia with sevoflurane (Mylan, Osaka, Japan). Motor function was observed daily and scored according to the following scales: Tail: 0 = no clinical sign; 1 = paralysis of the tail tip; 2 = incomplete paralysis of the entire tail; 3 = complete paralysis of the entire tail; forelimbs: 0 = no clinical sign; 1 = unable to climb fence using forelimbs; 2 = unable to walk; 3 = complete paralysis; left hind limb: 0 = no clinical sign; 1 = paralysis of the toe only; 2 = incomplete dorsiflexion of the foot joint while walking; 3 = complete paralysis (drags legs when walking). The total clinical score of disease severity (0–9 points) was calculated as the daily all-parts scores of rats in each group.

The number of rats used was 26, 21, 16, 10 and 5 for the subclinical, acute, peak, early recovery, and late recovery phases, respectively.

### Siponimod treatment

Siponimod (Selleck Biotech, Co., Ltd., Tokyo, Japan) was dissolved in 0.5% carboxymethylcellulose (CMC) in phosphate-buffered saline (PBS). Rats in the siponimod group were orally administered 1.0 mg/kg/day of siponimod from day 5 to 27 post-immunization (p.i.) daily. The EAN group, the non-treated control, received only CMC using the same protocol as the siponimod group.

### Tissue collection

On days 9, 12, 15, 21, and 28 p.i., rats anesthetized using sevoflurane were thoroughly perfused with ice-cooled PBS. Popliteal LNs and cauda equina (CE) were collected. CE at the second to third lumber spine levels was divided, fixed in 10% buffered formalin, and used for histological examinations. The remaining CE was stored in RNAlater™ (Qiagen, KK, Tokyo, Japan). The popliteal LNs were mechanically dissociated by passing through a 100 μm filter, and the resulting isolates were washed with Hanks' Balanced Salt Solution. After centrifuging at 1500 rpm for 5 min, and decanting the supernatant, the pellet was re-suspended in the medium at approximately concentration 10 million cells/ml. The lymphocytes, dissolved by RLT buffer (Qiagen, KK, Tokyo, Japan), and CE, preserved in RNAlater™ were stored at − 80 °C for later use to analyze gene expression.

### Histological analysis

Using CE from rat in the EAN and the siponimod groups on day 15 p.i, formalin-fixed CE were embedded in paraffin, cut into 5 μm-thick serial sections for Luxol fast blue (LFB) staining and counterstained with hematoxylin. Slides were assessed in a blinded manner for a myelinated area, characterized as LFB-positive without infiltration of inflammatory cells. The total myelinated area and the whole area of corresponding nerve roots were measured using the ImageJ software (National Institutes of Health, Bethesda, MD) after conversion to binary images (7–33 nerve roots; mean 15 roots for each rat). The proportion of myelinated area to the whole nerve root cross-section area was compared in both groups.

For immunohistochemistry for T cells and macrophages, anti-CD3 rabbit monoclonal antibody (SP7, Nichirei Biosciences Inc. Tokyo, Japan) for detection of T cells, anti-Iba-1 rabbit polyclonal antibody (GTX100042; GeneTex Inc., Irvine, CA) for detection of macrophages were applied on paraffin-embedded five µm-thick sections from rats of the EAN group or siponimod group on day 15 p.i. after standard antigen retrieval protocol recommended by the manufacturer.

To detect IFN-γ in CE from the EAN rats, anti-IFN-γ rabbit polyclonal antibody (Q69, Bioworld Technology, Inc., Bloomington, MN) was applied on paraffin-embedded five µm-thick sections from rats of the EAN group or siponimod group on day 12 p.i. without antigen retrieval protocol as recommended by the manufacturer.

To visualize these three first antibodies mentioned above on tissue sections, peroxidase-labeled anti-rabbit IgG goat polyclonal antibody conjugated with amino acid polymer (N-Histofine® Simple Stain MAX-PO (R)™, Nichirei Biosciences Inc. Tokyo, Japan) was applied followed by diaminobenzidine as a chromogen.

The CD3- or Iba-1-positive cells were detected to confirm the effect of siponimod on cellular infiltration in CE from rats of the EAN group or siponimod group on day 15 p.i. (*n* = 6). We counted the number of positive cells for CD3 or Iba-1 in entire cross-sections of CE at L3-4 levels. The total area of the same sections was measured using Image-J (National Institutes of Health, Bethesda, MD) to indicate these cell marker-positive cell densities (cell number per square millimeter).

To observe the cellular source of IFN-γ, sections from rats of the EAN group or siponimod group on day 12 p.i., namely the disease acute exacerbation phase, were stained by the above protocol.

### Sequential analysis of messenger RNA expression

Before extracting RNA, CE was crushed using a tissue grinder (Shakeman™, Biomedical Science, Tokyo, Japan). RNA extraction and cDNA synthesis were conducted using commercially available kits (RNeasy® Universal Mini kit, Qiagen, Tokyo, Japan; and iScript RT SuperMix for reverse transcription quantitative PCR, Bio-Rad, Tokyo, Japan). Next, cDNA was employed as the template for real-time PCR using iTaq™ Universal SYBR® Green Supermix (Bio-Rad) and gene-specific primers (Perfect Real Time Primer, TAKARA BIO, Otsu, Japan, and Bio-Rad). Sequencing primers are shown in Additional file 1: Table S1. The messenger RNA (mRNA) expression for cytokines and transcription factors related to EAN pathogenesis, Schwann cell (SC) generation, components of the myelin sheath, and peripheral nerve regeneration were analyzed semi-quantitatively and compared with that of the endogenous control using the CFX96 Touch™ Real-Time PCR Detection System (BIO-RAD). The gene expression levels presented as relative copy numbers using the delta threshold (2^−ΔΔCt^) method.

### Statistical analysis

For the clinical score, the collected data, reverse transcription PCR data between both groups were analyzed using the nonparametric Mann–Whitney *U*-test. Each statistical analysis was performed using the R software (version 3.2.2; R Foundation for Statistical Computing, Vienna, Austria) with the rms package [18]. A value of *p* < 0.05 was defined as statistically significant.

## Results

### The clinical course of EAN

**Fig. 1:**
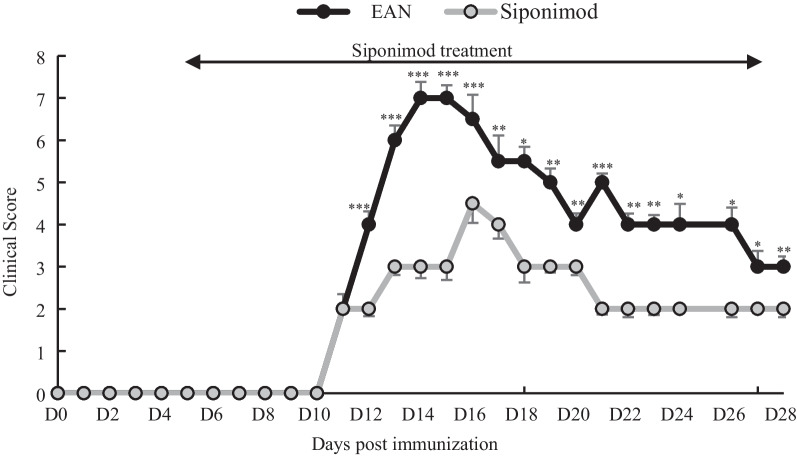
Siponimod effectively ameliorated clinical EAN. Temporal profile of motor impairment in rats. Decrease severity of flaccid paralysis was scored on a 0–9 scale. The siponimod group developed milder symptoms than that in the EAN group at all-scoring days. (*p < 0.05, **p < 0.01 or ***p < 0.001, EAN vs. Siponimod group, respectively). Data show mean ± SEM. Statistics analysis using Mann-Whitnry *U*-test

Fifty-two rats were divided into the EAN group, which was given only CMC (*n* = 26), and the siponimod group (*n* = 26). All rats in both groups developed flaccid paralysis of the tail and limbs on days 10–11 p.i. In the EAN group, paralysis progressed rapidly, resulting in quadriparesis on days 13–15 p.i., and gradually improved spontaneously from day 16 p.i. onwards. The severity in the siponimod group was significantly milder than in the EAN group through the entire period after symptom development, such as the exacerbation, peak, and recovery phases (*p* < 0.05, *p* < 0.01, *p* < 0.001, respectively) (Fig. 1).

Based on these results, we defined the subclinical phase as a period from the day of immunization to the day before the onset of symptoms (day 0–9 p.i.), the exacerbation phase as a period in which minimal to moderate motor impairment occurs (day 10–12 p.i.), the peak phase, as the period of worst motor impairment (day 13–15 p.i.), and the recovery phase as the period starting at the time when symptoms began to improve until the last day of observation (day 16–28 p.i.).

### Histological examination of cauda equina

**Fig. 2:**
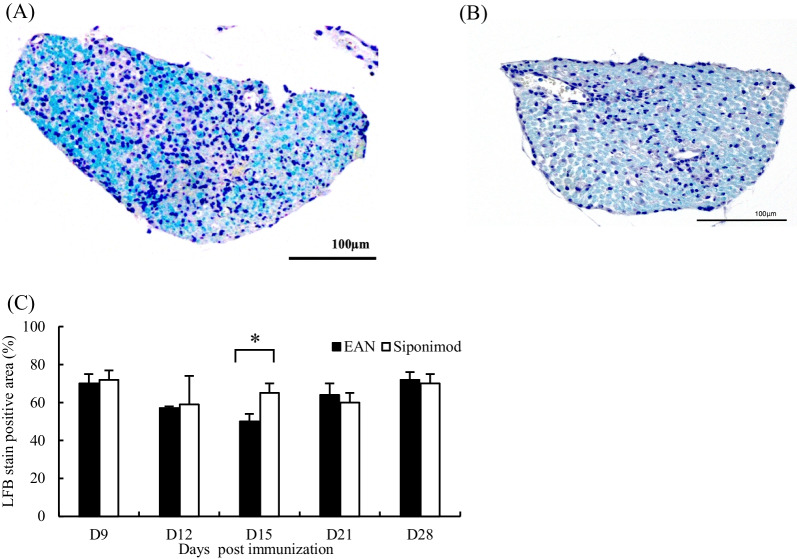
Histological examination of cauda equina. **A** Patchy mononuclear cell infiltration foci are seen on the cauda equina (CE) nerve of rat in the EAN group on day 15 after immunization (p.i.). **B** CE nerve of rat in the siponimod group on day 15 p.i. Lesser cell infiltration and more LFB-positive area preserved normal myelination than that of rat in the EAN group. **C** The siponimod group showed statistically significant suppression of demyelination compared with that in the EAN group on day 15 p.i. which is the peak phase of EAN. (Data show mean ± SEM. * p < 0.01, EAN vs. siponimod groups. Statistics analysis using Mann–Whitney *U*-test.). However, there was no statistically significant difference on days 9, 12, 21, and 28 p.i.

On day 15 p.i., mononuclear cell infiltration, which appeared patchy and multifocal, was observed in both groups (Fig. 2A and B), whereas CE nerve from the rat in the siponimod group (Fig. 2B) showed less demyelination and inflammatory cell infiltration in the region compared with that from the EAN group (Fig. 2A).

Sequential examination of LFB-stained sections of CE revealed that the myelinated area was significantly preserved in the siponimod group compared with that in the EAN group at day 15 p.i. (Fig. 2C, the EAN group; 48.9 ± 4.2%; the siponimod group, 66.1 ± 4.2% *p* < 0.01). However, there were no significant differences between both groups in other time points, viz., days 9, 12, 21, and 28 p.i..

**Fig. 3:**
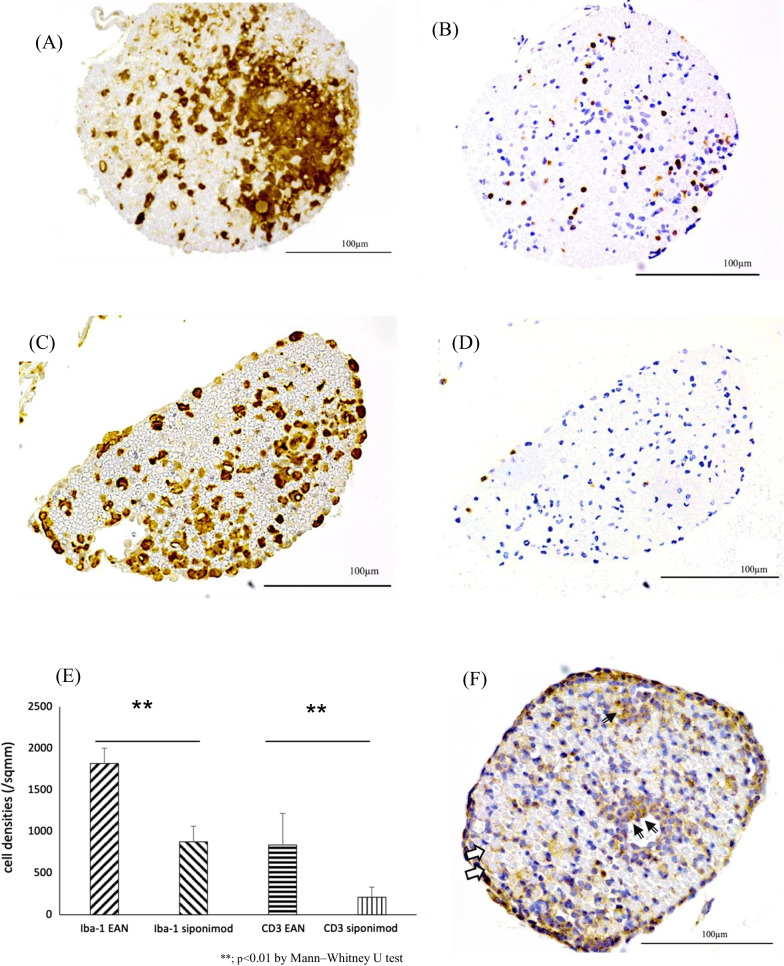

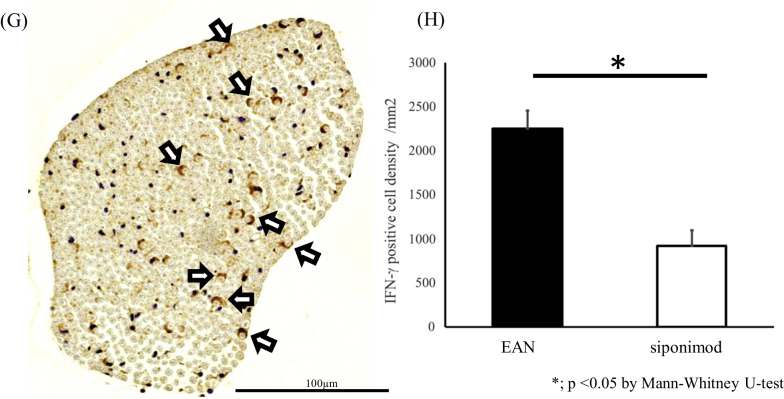
Immunohistochemical study of cauda equina. Serial sections of the CE from rats in the EAN or siponimod group on day 12 or15 p.i were stained for the expression of Iba-1 (macrophages), CD3 (T lymphocytes), or IFN-γ using a standard immunohistochemistry protocol, with hematoxylin counterstain. The bars indicate 100 µm. **A** Iba-1 staining of the CE from the EAN group rat exhibits dense macrophage infiltration, primarily in the vicinity of the endoneurial vessel, spreading to the entire endoneurium. **B** A serial section stained for CD3 revealed numerous T cells infiltrating the endoneurium, although they were fewer than the macrophages. **C** Iba-1 staining in the siponimod group rat shows fewer macrophage infiltration than in the EAN group rat. **D** CD3 staining indicates that T cells are much fewer in the siponimod group rat than in the EAN group rat. **E** The number of these cells in the entire cross-sections of the CE in each sample was counted, and the corresponding cross-sectional areas were measured to determine the cell density in each group. The lower density of macrophages and T lymphocytes infiltration was observed in the siponimod group rats than in the EAN group rats (**; *p* < 0.01: EAN vs. siponimod groups. Statistics analysis using Mann–Whitney *U*-test.). **F**. Immunostaining of IFN-γ in CE of the EAN group rat and **G** the siponimod group rat on day 12 p.i.. Mononuclear cells producing IFN-γ were fewer in the siponimod group rat (black arrows in **F**) than in the EAN group rat. Crescent-shaped IFN-γ positive cells were found in both groups (white arrows in **F** and **G**). Those were more frequent in the siponimod group. **H** Comparison of the density of IFN-γ expressing mononuclear cells in CE between the EAN group and the siponimod group on day 12 p.i.. Significantly fewer IFN-γ-expressing cells were observed in the siponimod group. (*p < 0.05: EAN vs. siponimod group. Statistics analysis using Mann–Whitney *U*-test.)

Immunohistochemical analysis of macrophages and T lymphocytes infiltrated CE nerve of the siponimod group rat on day 15 p.i. demonstrated a lower number of Iba-1-positive macrophages and CD3-positive T lymphocytes compared to those in the EAN group rat (Fig. 3C and D vs. Figure 3A and B, respectively). Statistical analysis revealed that the density of Iba-1-positive and CD3-positive cells in the CE nerve of the siponimod group was significantly lower than that of the EAN group (876.2 ± 188.3/mm^2^ vs. 1815.9 ± 186.2/mm^2^, and 211.7 ± 122.2/mm^2^ vs. 836.5 ± 381.7/mm^2^, *p* < 0.01 and *p* < 0.01, respectively; Fig. 3E).

There were two types of IFN-γ producing cells observed in CE on day 12 p.i.. Many of them were mononuclear cells with round-shaped nuclei localized in perivascular infiltrating cell foci (black arrows in Fig. 3F). The remains were crescent-shaped cells in contact with myelinated nerve fibers, presumably SCs (white arrows in Fig. 3F and G). Crescent-shaped IFN-γ -positive cells were found in the siponimod group more frequently than in the EAN rats (white arrows in Fig. 3G).The density of IFN-γ expressing cells with round-shaped nuclei of CE nerve in the siponimod group was significantly lower than that of the EAN group (922.7 ± 176.9 cells /mm^2^ vs. 2248.5 ± 211.4 cells/mm^2^, *p* < 0.05, Fig. 3H)

### The mRNA expression of EAN pathogenesis-related molecules

**Fig. 4:**
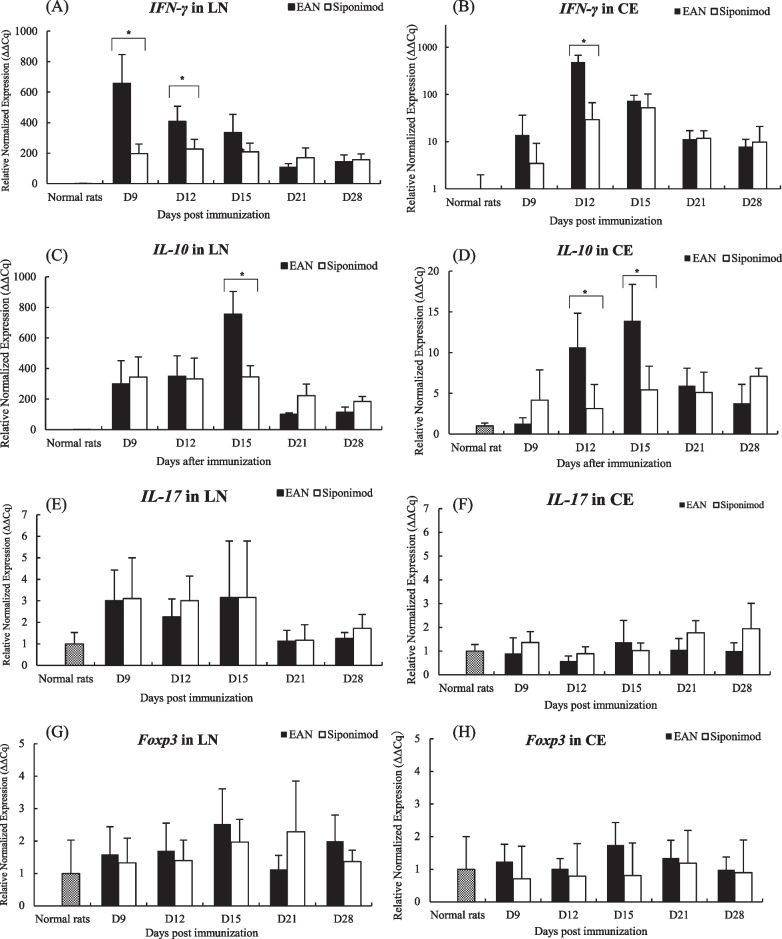
Siponimod affects the sequential expression of cytokines and transcription factors mRNAs expression in the popliteal lymph nodes and the cauda equina. The bar graphs show the expression of IFN-γ, IL-10, IL-17, and Foxp3 mRNAs in the popliteal lymph nodes (LN) and in the cauda equine (CE) at days 9, 12, 15, 21, and 28 p.i.. Data for **A** IFN-γ, **C** IL-10, **E** IL-17, and **G** Foxp3 were data of LN. **B** IFN-γ, **D** IL-10, **F** IL-17, and **H** Foxp3 mRNAs in CE. Data show mean ± SEM. *p < 0.05: EAN vs. siponimod groups. Statistics analysis using Mann–Whitney *U*-test

The IFN-γ mRNA expression increased in both groups from the subclinical to the peak phases (Fig. 4A and B). Those were lower in the siponimod group at the subclinical phase of LN and CE and in the subclinical and acute phase of CE than EAN (*p* < 0.05, *p* < 0.05, and *p* < 0.05, respectively). The IL-10 mRNA expression increased and peaked following IFN-γ from the subclinical to peak phase (Fig. 4C and D). Those were lower in the siponimod group at the subclinical phase of CE and the subclinical and exacerbation phases of LN and CE than in EAN (*p* < 0.05, *p* < 0.05, and *p* < 0.05, respectively). The IL-17 and Foxp3 mRNA expression in LN and CE from both groups did not change compared to the normal rats’ group at all phases (Fig. 4E–H).

### The mRNA of the molecules in the cauda equine-related expressed during the peripheral nerve repair process

**Fig. 5:**
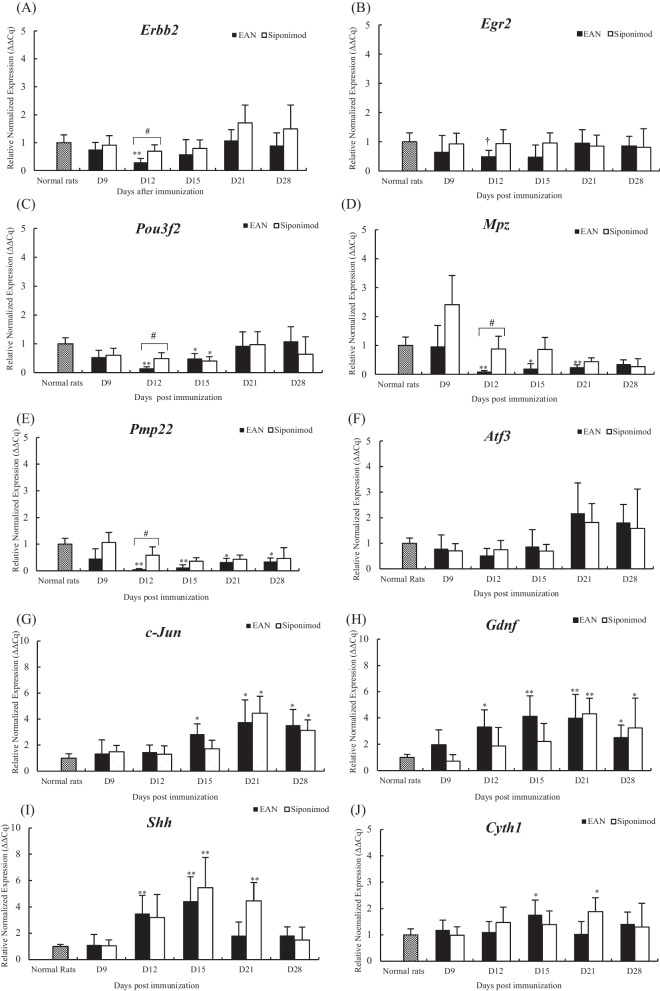
Sequential change of mRNA expression of the molecules related to demyelination, repair program, and remyelination during peripheral nerve injury in cauda equina. The sequential changes in Erbb2, Egr2, Pou3f2, Mpz, Pmp22, Atf3, c-Jun, Gdnf, Shh, and Cyst1 mRNAs in the cauda equina are shown in the bar graphs. Data for **A** Erbb2, **B** Egr2, **C** Pou3f2, **D** Mpz, **E** Pmp22, **F** Atf3, **G** Gdnf, **H** Shh, and **I** Cyth 1 inCE. Data show mean ± SEM. †*p* < 0.1: Normal rats vs. EAN, *p < 0.05, **p < 0.01: Normal rats vs. EAN or siponimod groups, #*p* < 0.05: EAN vs. siponimod groups, Statistics analysis using Mann–Whitney *U*-test

The mRNA expression of SC generating factors’, receptor tyrosine-protein kinase 2 (Erbb2), early growth response 2 (Egr2), and POU class 3 homeobox 2 (Pou3f2), decreases at the exacerbation phase, but the degree of decrease was milder in the siponimod group than in the EAN group (*p* < 0.05, *p* < 0.1, and *p* < 0.05, respectively) (Fig. 5A–C). The mRNA expression of components of myelin sheaths, myelin protein zero (Mpz) and peripheral myelin protein 22 (Pmp22), in both groups decreased from the exacerbation to the recovery phases. Nonetheless, that degree of decrease at the exacerbation phase was milder in the siponimod group than in the EAN group. (*p* < 0.05, and *p* < 0.05, respectively) (Fig. 5D, E). The mRNA expression of peripheral nerves regenerating factors’, activating transcription factor 3 (Atf3), Jun proto-oncogene (c-Jun), glial cell line-derived neurotrophic factor (Gdnf), sonic hedgehog signaling molecule (Shh), and cytohesin 1 (Cyth1) increased from the peak to the recovery phases but was not different in the two both groups (Fig. 5F–J).

S1PR1 and S1PR5 are expressed in SCs [19]. Additional file 1: Figure S1 in Supplemental data shows the mRNA expression of S1PR5 in CE, including that in SCs from normal rats, and from those in the EAN and siponimod groups. Expression of S1PR5 mRNA did not correlate with protein expression, but there was no significant difference among the three groups during the clinical course.

## Discussion

In this study, we found that siponimod improved the clinical course of EAN accompanied by inhibition of T cells and macrophages infiltration and suppression of demyelination in CE. The main effect of siponimod in cytokine milieu was a decrease in the expression of IFN-γ and IL-10 mRNAs in LN and CE, with little effect on SC regeneration markers.

Siponimod at 1.0 mg/kg/day did not completely inhibit the EAN development, whereas the same dose of fingolimod reportedly suppressed it [14]. This difference may be attributable to the timing of treatment initiation, disease susceptibility and severity, pharmacokinetics, and immunization protocols. The efficacy of 3 mg/kg siponimod has been reported in experimental autoimmune encephalomyelitis [20]. The efficacy of 3 mg/kg siponimod for EAN should be studied in the future. T-helper 1 and Th2 cells play a crucial role in the pathogenesis of EAN, and there are reports on the involvement of some Th17 cells [21]. IFN-γ produced from Th1 cells increases rapidly from the subclinical phase to the peak phase, and IL-10 production from Th2 cells increases from the peak phase to convalescent phase [22]. In this study, expression of IFN-γ and IL-10 mRNA showed the same trend as in this report. Siponimod prevented the egress of activated lymphocytes, resulting in a decrease in IFN-γ mRNA expression in CE. Interestingly, the expression of IFN-γ mRNA in LN was also decreased. The inhibition of auto-reactive T cell egress from LN and stagnation of many T cells in the T cell zone might lead to the suppression of proliferation or IFN-γ production.

The IL-10 strongly inhibits the production of IL-12 and IL-23 by antigen-presenting cells, thereby impairing their ability to promote the differentiation and maintenance of Th1 and Th17 cells that produce IFN-γ and IL-17, respectively [23]. We observed reduced expression of IL-10 mRNA in the siponimod group compared with that in the EAN group during the CE exacerbation and peak phases, which suggests that IL-10 production is probably dependent on the need to suppress inflammation by macrophages induced by IFN-γ. Therefore, it is reasonable to assume that the low expression of IL-10 in the siponimod group is a consequence of the low expression of IFN-γ. Another possibility is that siponimod impedes the migration of not only IFN-γ-producing T cells, but also of IL-10-producing T cells, from the LNs to the CE. This may be responsible for the slowly ameliorating course of EAN symptoms in the siponimod group, despite the expression of IL-10.

The molecular mechanisms involved in SCs plasticity have been studied using models of peripheral nerve transection and compression injury [24]. Conversely, EAN is a model of the autoimmune-associated neuritis. The pathological findings show extensive inflammatory demyelinating lesions in the ventral spinal nerve roots and sciatic nerve, in addition to the axonal damage [17, 25]. To the best of our knowledge, this is the first report on the sequential changes in molecules that contribute to nerve regeneration in the inflammatory nerve injury model.

After the nerve injury, highly differentiated and specialized myelinating SCs are reprogrammed into proliferative progenitor-like repair SCs that drive the entire regeneration process [26, 27]. Reprogramming of SC under physiological conditions is mainly defined as de-differentiation but repaired SC re-expresses not only immature SC markers but also exhibits completely different characteristics. Therefore, it is thought to be more appropriate to describe it as trans-differentiation (i.e., evolving into a distinct phenotype) [28]. Transcription factor c-Jun is downregulated after birth during SC differentiation and myelination and is highly upregulated under pathological conditions such as peripheral nerve injury, demyelinating diseases, and other peripheral neuropathies [29–31]. The expression of c-Jun mRNA in EAN increased from the peak phase to the recovery phase, and then this factor was also involved in the recovery of injured peripheral nerves by the autoimmune neuritis.

The C-Jun is a negative regulator of myelination and an antagonist of Egr2, one of the main pro-myelinating transcription factors, and c-Jun and Egr2 expression is reciprocally exclusive.

The expression of c-Jun and Erg2 mRNAs expression did not necessarily show opposite trends, but at least Egr2 was not elevated during the recovery period when c-Jun was elevated.

Immature SCs produce relatively high levels of c-Jun and low levels of Egr2, whares myelinating cells switch from high levels of Egr2 to low levels of c-Jun [32, 33]. Moreover, the decrease in expression of Mpz and Pmp22 mRNAs is consistent with the process associated with the SC trans-differential. This suggests that demyelination and secondary axonal damage by the macrophages and inflammatory-related cytokines induced trans-differentiation of mature SC to immature SC, which produced c-Jun, from the worsening phase to the recovery phase in EAN.

On the contrary, the expression of Egr2, Mpz, and Pmp22 mRNAs did not increase until the end of the clinical course, supporting the notion the myelinating SCs had not yet fully regenerated. Interestingly, the flaccid paralysis in the EAN and siponimod groups peaked on day14–16 p.i. and began to improve thereafter. This suggests that factors other than the regeneration of the myelin sheath are responsible for the recovery of symptoms.

In CIDP, oxidative burst is significantly increased in fresh whole blood from the patients [34]. N(epsilon)-carboxymethyllysine, a biomarker for oxidative stress, was found in peripheral neurons in some patients with CIDP [35]. In our previous study, the increase in the hydroxyl radicals in CE of EAN rats is maximal between the exacerbation to the peak phases [36]. The reactive oxygen species cause a chain reaction involving lipid peroxidation, which secondarily damages the normal lipid-rich SCs. The metabolites that indicate the degree of lipid oxidation also increase at the peak phase [37]. Both products return to pre-immunized levels after the peak of symptoms is achieved. These factors may be the other possible explanations for the improvement of clinical symptoms of EAN before the completion of remyelination.

Furthermore, Shh has been reported to directly affect attracting macrophages in pathologic conditions associated with tissue damage other than peripheral neuropathy [38, 39]. In the inferior alveolar nerve transection experiments, hedgehog pathway inhibitors reduced macrophage infiltration and prolonged the duration of injured myelin basic protein attached to the nerve [40]. This finding suggests that increased expression of Shh mRNA may contribute to the clearing of damaged axons and SC debris by the induction of macrophages in EAN. Damaged axons and SC fragments should interfere with the velocity of nerve conduction; therefore, the early removal of this debris should be one of the causes of the improvement of motor function in EAE without SC maturity.

The Gdnf belongs to the TGF-βfamily of neurotrophic factors and performs versatile and distinct roles in neuronal signaling pathways. Elevation of Gdnf in the sciatic nerve was reported in different axonal injury models [40]. The chronic injury to the adult rat sciatic nerve induces a rapid up-regulation of Gdnf mRNA in SCs proximal as well as distal to the injury site, and that remains at high levels for at least 5 months after injury [41–43]. In our study, the increase in the expression of Gdnf mRNA was monomodal by the clinical course of EAN, suggesting that axonal damage, if any, in EAN is transient. Siponimod triggers S1PR1-dependent anti-inflammatory effects on pathogenic lymphocytes and glial cells in the CNS, and S1PR5-dependent pro-repair effects on oligodendrocytes, sparing S1PR3/4-dependent pro-inflammatory effects on astrocytes [44]. In vitro and in CNS of tadpoles of the African clawed frog, siponimod has been shown to promote oligodendrocyte remyelination via the S1PR5 [45, 46]. Siponimod did not increase the levels of molecules that promote remyelination in treated group compared with that in the EAN group. This may be because the effect of siponimod is due mainly to the inhibition of effector T cell infiltration into CE via S1PR1. Selective S1PR5 agonists [47] will be necessary to investigate S1PR5-mediated effects on peripheral nerve injury in EAN.

There are several limitations to this study. First, the dose of siponimod in this experimental therapeutic study was much higher than the dose usually used for human patients when converted to an amount per body weight. Therefore, the same dose cannot be applied directly to human patients. Secondly, since the target antigens and detailed pathological mechanisms in cellular immunity have not been clarified in human autoimmune peripheral neuropathy, there is a question as to whether the effects seen in the EAN study can be achieved in humans. However, despite these limitations, it is exciting to note that it may lead to the development of new CIDP therapies.

## Conclusion

Siponimod has a potential to ameliorate EAN. Shh and Gdnf, as well as c-Jun, play a significant role during the process of recovery of injured nerves.

## Supplementary Information

**Additional file 1: Table S1.** Primer sequences used in real-time PCR. **Figure S1.** Expression of S1PR5 mRNA in cauda equina.

## Data Availability

The data sets supporting the findings of this study are available from the corresponding author upon reasonable request.

## Abbreviations

EAN: Experimental autoimmune neuritis
S1PR: Sphingosine-1-phosphate receptor
GBS: Guillain–Barré syndrome
EAE: Experimental autoimmune encephalomyelitis
mRNA: Messenger ribonucleic acid
CNS: Central nervous system
CMC: Carboxymethylcellulose
PBS: Phosphate-buffered saline
LN: Lymph node
CE: Cauda equina
HE: Hematoxylin eosin
LFB: Luxol fast blue
IFN-γ: Interferon gamma
IL-10: Interleukin-10
IL-17: Interleukin-17
Foxp3: Forkhead box protein P3
SC: Schwann cell
Erbb2: Receptor tyrosine-protein kinase 2
Egr2: Early growth response 2
Pou3f2: POU class 3 homeobox 2
Mpz: Myelin protein zero
Pmp22: Peripheral myelin protein 22
c-Jun: Jun proto-oncogene
Gdnf: Glial cell line-derived neurotrophic factor
Atf3: Activating transcription factor 3
Shh: Sonic hedgehog signaling molecule
Cyth1: Cytohesin 1
