# Supplementary material for: Siponimod ameliorates experimental autoimmune neuritis
Source: J Neuroinflammation. 2023 Feb 14;20:35. doi: 10.1186/s12974-023-02706-z (PMC9926865; doi:10.1186/s12974-023-02706-z)
Supplement: Supplementary file 1 — Additional file 1: Table S1. Primer sequences used in real-time PCR. Figure S1. Expression of S1PR5 mRNA in cauda equina. [file 12974_2023_2706_MOESM1_ESM.docx]

**Supplemental information**

**Table 1. Primer sequences used in real-time PCR**

| Gene name | Primer | Sequence (5′-3′) |
| --- | --- | --- |
| *Gapdh* | Forward | GGCACAGTCAAGGCTGAGAATG |
|  | Reverse | ATGGTGGTGAAGACGCCAGTA |
| *IFN-γ* | Forward | CATCACAGCAACAACATAAGTGTCATC |
|  | Reverse | CATTGACAGCTTTGTGCTGGA |
| *IL-10* | Forward | CAGACCCACATGCTCCGAGA |
|  | Reverse | CAAGGCTTGGCAACCCAAGTA |
| *IL-17* | Forward | CTGATCAGGACGAGCGACCA |
|  | Reverse | ACTGTAGCCTCCAGGTTCAGTAGCA |
| *Foxp3* | Forward | TGAGCTGGCTGCAATTCTGG |
|  | Reverse | ATCTAGCTGCTCTGCATGAGGTG |
| *Erbb2* | Forward | CTGGAAGTACCCGGATGAGGA |
|  | Reverse | GCCCACTACAGTTGCAATGATGA |
| *Erg2* | Forward | AATGGCTTGGGACGGACTTG |
|  | Reverse | ACATATTGCTTAAGGGCTGAGATGG |
| *Pou3f* | Forward | GGCACTGTGAACAGACCTGGAA |
|  | Reverse | AAGCTACCAGAGCAGCTTGAGGAC |
| *Pmp22* | Forward | ATCTCTGGCAGAACTGTACCACATC |
|  | Reverse | ATCATGGTGGCCTGGACAGA |
| *Atr3* | Forward | GCTGCTGCCAAGTGTCGAA |
|  | Reverse | CGGTGCAGGTTGAGCATGTA |
| *c-Jun* | Forward | GGGAACAGGTGGCACAGCTTA |
|  | Reverse | ACGTTTGCAACTGCTGCGTTA |
| *Shh* | Forward | AGGCTGGATTCGACTGGGTCTA |
|  | Reverse | AACTTGGTGCCACCCTGCTC |
| *Cyth1* | Forward | CTTTGCGCAGCGGTACTGTC |
|  | Reverse | TCTCCACCGTAGGCTTGTCTTTG |
| *Gdnf* | Amplicon Context Sequence* | ACTGATCTTTCGATATTGTAGCGGTTCCTGTGAAGCGGCCGAGACAATGTACGACAAAATACTAAAAAATCTGTCTCGAAGTAGAAGGCTAACAAGTGACAAGGTAGGCCAG |

*Primer sequence is not available.

**Figure 1. Expression of S1PR5 mRNA in cauda equina**
